# The Lock-Jaw of Infants

**Published:** 1884-11

**Authors:** 


**The Lock-Jaw of Infants** (Trismus Nascentium); or, Nine-day Fits, Crying-spasms, etc. Its history, cause, prevention and cure. By J. F. HARLIGAN, M. D., Washington, D. C., Member of the American Medical Association, etc. Bermingham & Co., 28 Union Square, New York; 14 Cockspur Street, Pall-Mall, London. 1884. Price, 75c.

The author gives a very complete history of this very strange disease of infancy, with all the conflicting theories as to its causation. He then gives his own opinion and says that the disease is the result of pressure exerted at the base of the brain by inward displacement of the occiput, differing in degree from the slightest to the greatest. There is yet another class of cases which he describes, plainly caused by an opposite state of things, namely, prolonged lateral decubitus, with a position of the occiput exterior to the parietal bones. The author illustrates his work with the citation of cases, with table showing the influence of age, color, seasons, etc., the correction of the displacement of the occiput by changing its position so as to relieve the pressure from the overriding of the occiput, the replacing of the displaced bones being the treatment advised, based upon the cause, which the author has endeavored to elucidate. The work is a valuable contribution to the literature of trismus. We have read it with pleasure and profit, and commend it to those interested in diseases of children.

